# Thoracoscopic complex pulmonary basal subsegmentectomy: A combined subsegmentectomy of left s9b+10b

**DOI:** 10.1016/j.xjtc.2021.12.012

**Published:** 2022-01-21

**Authors:** Chengwu Liu, Wenping Wang, Lunxu Liu

**Affiliations:** a Department of Thoracic Surgery, West China Hospital, Sichuan University, Chengdu, China; b Western China Collaborative Innovation Center for Early Diagnosis and Multidisciplinary Therapy of Lung Cancer, Sichuan University, Chengdu, China

**Figure:**
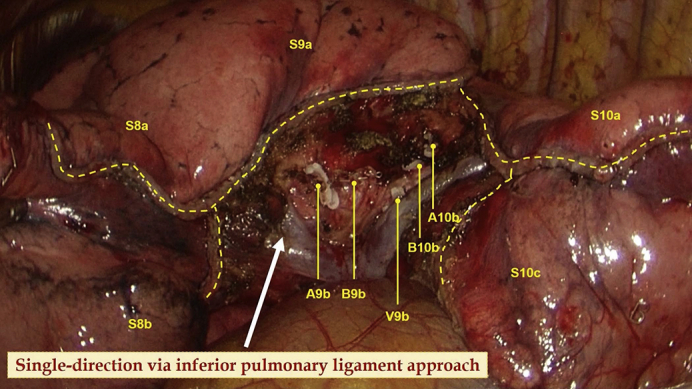
The single-direction thoracoscopic combined basal subsegmentectomy of left S9b+10b.

**Central Message**

The single-direction method allows complex thoracoscopic combined basal subsegmentectomy such as Left S9b+10b to be performed successfully.

See Commentary on page 210.

Segmentectomy is widely used in the treatment of peripheral pulmonary nodules while preserving more functional pulmonary parenchyma.^1^ For intersegmental nodules, which are deeply located in the pulmonary parenchyma near the intersegmental border, a sufficient margin might not be obtained from a simple segmentectomy. In such cases, combined subsegmentectomy becomes the appropriate therapy of choice.^2^ As thoracoscopic anatomical basal segmentectomies, those involving the lateral (S9) and/or posterior (S10) basal segments are technically challenging,^3^ and combined subsegmentectomy involving S9b and S10b would be one of the most technically demanding ones. To the best of our knowledge, there has been no report on it. Herein, we aimed to share a case of combined subsegmentectomy (left S9b+10b) to introduce techniques to address a complex combined basal subsegmentectomy.

## Case Presentation

A 58-year-old woman was referred to us for a ground-glass opacity (GGO) nodule (9 × 6 mm) in the left lower lobe revealed by the high-resolution computed tomography (HRCT). No radiologic changes had occurred at the 3-month follow-up. Therefore, the patient was referred for thoracoscopic surgery. Informed written consent and institutional review board approval were both obtained (No. 2021-879; July 20, 2021) for the surgery and the publication of the study data.

**Figure 1:**
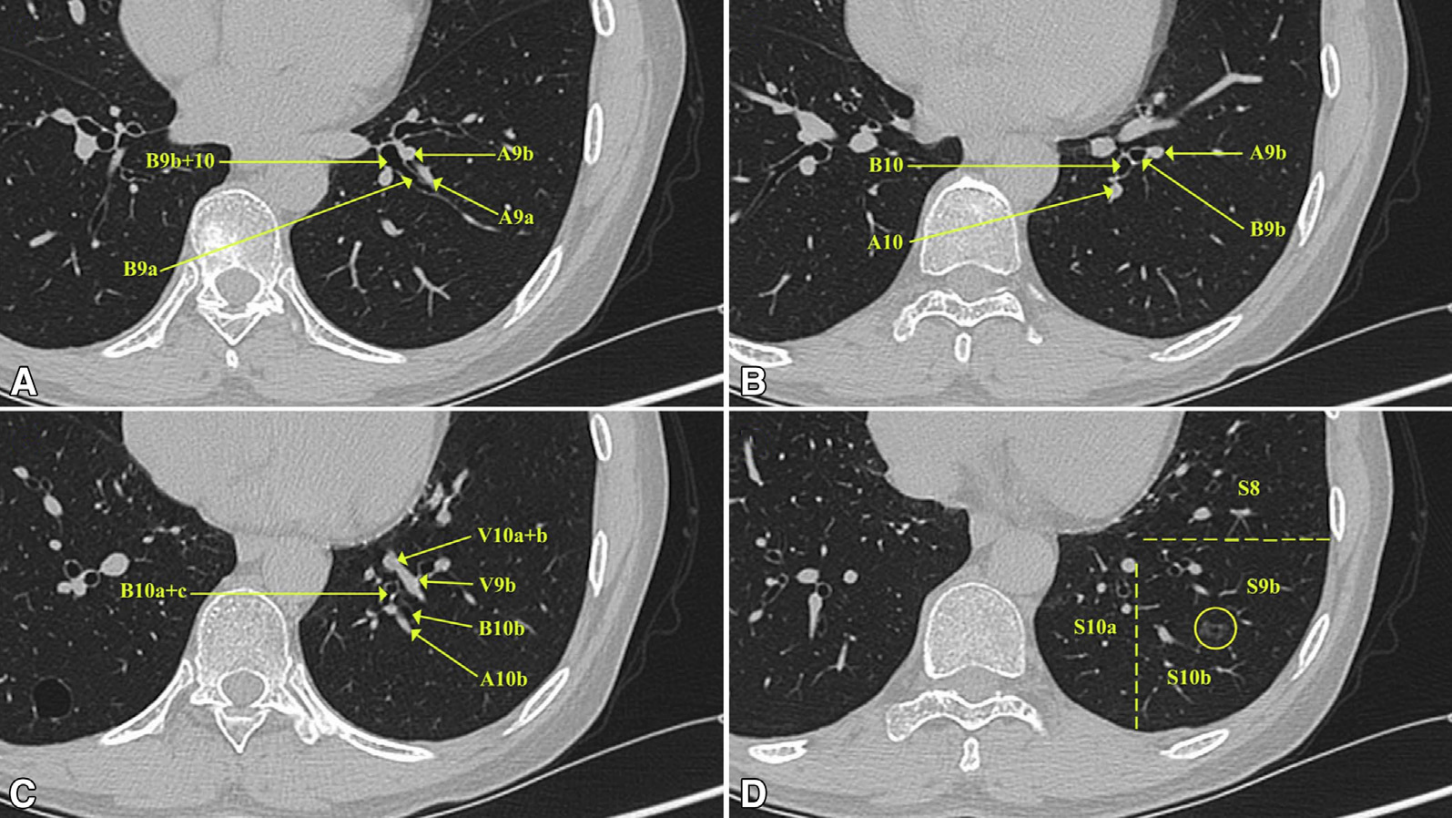
Identifying the subsegmental structures of the left basal segment and their positional relations on preoperative HRCT. A, Identifying B9a and A9a. B, Identifying B9b and A9b. C, Identifying B10b, A10b, and the intersubsegmental vein (V9b). D, Showing the lesion located near the intersubsegmental border between S9b and S10b.

The surgical plan was made mainly based on the preoperative HRCT (Figure 1, *A-C*). A combined subsegmentectomy (S9b+S10b) was planned because the nodule was located just near the intersegmental border between S9b and S10b (Figure 1, *D*).

**Figure 2:**
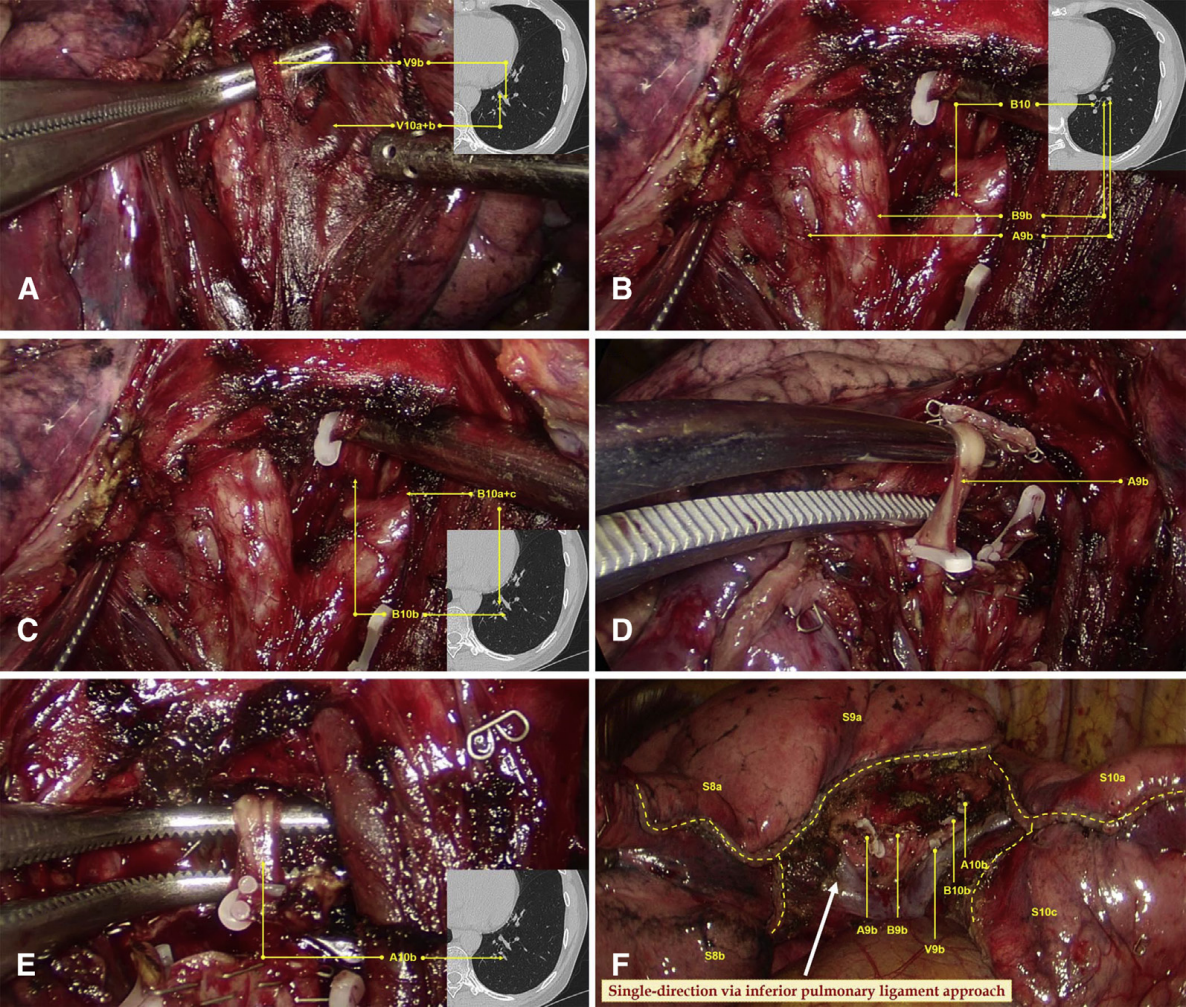
The single-direction combined basal subsegmentectomy of left S9b+10b through the inferior pulmonary ligament approach. A, Dissecting the inferior pulmonary vein and identifying the intersubsegmental vein of V9b. B and C, Dissecting the basal segmental bronchus and identifying B9b and B10b. D, Dissecting and managing A9b just posterior to B9b. E, Identifying and managing A10b just posterior to B10b. F, Showing the stumps after complete resection of left S9b+10b.

The details of the procedures are introduced in Video 1. The surgery was initiated by dissecting the inferior pulmonary ligament and then the basal segmental vein and its branches. After that, the procedure proceeded in a single-direction manner from the caudal side to the cranial side and from the superficial to deep structures. The target subsegmental structures of V9b (Figure 2, *A*), B9b and B10b (Figure 2, *B* and *C*), A9b (Figure 2, *D*), and A10b (Figure 2, *E*) were dissected and transected in order of appearance. After that, the intersubsegmental demarcation line was identified by the modified inflation–deflation method and the intersubsegmental planes were managed using stapler-based tailoring method. Finally, the thoracoscopic combined basal subsegmentectomy of LS9b+10b was completed (Figure 2, *F*). Intraoperative frozen section pathologic examination documented an adenocarcinoma. Lobe-specific lymph node sampling was performed, with stations 7, 9, 10, and 13 lymph nodes removed.

**Video 1:**
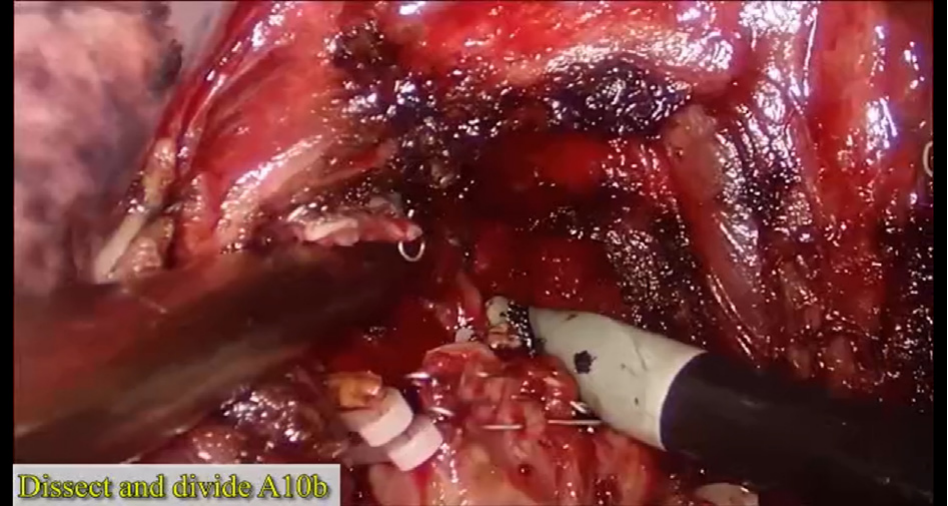
A 3-port thoracoscopic left S9b+10b combined subsegmentectomy. The operation was performed through the inferior pulmonary approach following the concept of single-direction. The nomenclature of subsubsegments is adopted according to the Japanese Committee on the Nomenclature for Bronchial Branching. *VATS*, Video-assisted thoracic surgery; *CT*, computed tomography; *HRCT*, high-resolution computed tomography; *GGN*, ground-glass nodule; *POD*, postoperative day; *AIS*, adenocarcinoma in situ. Video available at: https://www.jtcvs.org/article/S2666-2507(22)00025-6/fulltext.

The operation time was 130 minutes, and the intraoperative blood loss was 20 mL. The postoperative course was uneventful, and the patient was discharged on postoperative day 4. The final pathologic examination documented a 9-mm adenocarcinoma in situ with negative surgical margin, pTisN0M0.

## Comment

For GGO-dominant lung cancers, both wedge resection and anatomic segmentectomy can provide an excellent prognosis if sufficient margins are obtained and the 2 procedures are mainly selected based on the site of the lesion.^4^ Sometimes, the lesion may present just beside the intersegmental border between 2 segments. Under such circumstance, resection of either segment is not enough to provide a safe margin. In this case, the lesion was a subcentimeter pure GGO located just beside the intersubsegmental border between S9b and S10b. An early-stage pulmonary adenocarcinoma was highly suspected according to the radiologic features, and surgery was indicated. After carefully analyzing the HRCT, we found that a combined subsegmentectomy of S9ba and S10b could provide a safe margin and help preserve the functioning pulmonary parenchyma of S9a and S10a+c. Therefore, we chose to perform a combined subsegmentectomy for this patient.

It is especially challenging to perform basal segmentectomies because segmental structures are deeply located in the parenchyma, anatomic variations are common, and division of the intersegmental planes is difficult. As is commonly found, S9b and S10b are the 2 innermost subsegments of the basal segment either on the right side or the left side. In addition, the 2 subsegments are facing the diaphragmatic surface. It's hard to approach the subsegmental hili of S9b and S10b through the interlobar fissures. We have previously described the technical procedures of single-direction thoracoscopic basal segmentectomies using the transinferior ligament approach and the stem-branch method in detail.^3^^,^^5^ Our method can be used for all kinds of basal segmentectomies, including S9 segmentectomy, which is considered as the most challenging one. However, this case was much more complex because the 2 subsegmental hili needed to be managed and the target structures were much more deeply located. After dissecting the inferior pulmonary ligament, we divided S8 and S10 along the intersegmental septum between them. Only after that could we obtain access to the subsegmental hili of S9b and S10b. Then, we dissected along the stems of the basal bronchi and vasculatures, tracked their branches, and identified them according to their positional relationships obtained from preoperative planning using the method of stem-branch. Finally, we successfully completed this complex combined basal subsegmentectomy.

Consequently, thoracoscopic combined basal subsegmentectomy of LS9b+10b can be performed through the inferior pulmonary ligament approach by using the method of stem-branch in a single-direction manner.

## Supplemental Data

Video 1 A 3-port thoracoscopic left S9b+10b combined subsegmentectomy. The operation was performed through the inferior pulmonary approach following the concept of single-direction. The nomenclature of subsubsegments is adopted according to the Japanese Committee on the Nomenclature for Bronchial Branching. *VATS*, Video-assisted thoracic surgery; *CT*, computed tomography; *HRCT*, high-resolution computed tomography; *GGN*, ground-glass nodule; *POD*, postoperative day; *AIS*, adenocarcinoma in situ. Video available at: https://www.jtcvs.org/article/S2666-2507(22)00025-6/fulltext.
